# A robotic arm control system with simultaneous and sequential modes combining eye-tracking with steady-state visual evoked potential in virtual reality environment

**DOI:** 10.3389/fnbot.2023.1146415

**Published:** 2023-03-27

**Authors:** Rongxiao Guo, Yanfei Lin, Xi Luo, Xiaorong Gao, Shangen Zhang

**Affiliations:** ^1^School of Integrated Circuits and Electronics, Beijing Institute of Technology, Beijing, China; ^2^Department of Biomedical Engineering, School of Medicine, Tsinghua University, Beijing, China; ^3^School of Computer and Communication Engineering, University of Science and Technology Beijing, Beijing, China

**Keywords:** steady-state visual evoked potentials (SSVEP), eye-tracking, hybrid brain-computer interface (h-BCI), robotic arm, virtual reality (VR)

## Abstract

At present, single-modal brain-computer interface (BCI) still has limitations in practical application, such as low flexibility, poor autonomy, and easy fatigue for subjects. This study developed an asynchronous robotic arm control system based on steady-state visual evoked potentials (SSVEP) and eye-tracking in virtual reality (VR) environment, including simultaneous and sequential modes. For simultaneous mode, target classification was realized by decision-level fusion of electroencephalography (EEG) and eye-gaze. The stimulus duration for each subject was non-fixed, which was determined by an adjustable window method. Subjects could autonomously control the start and stop of the system using triple blink and eye closure, respectively. For sequential mode, no calibration was conducted before operation. First, subjects’ gaze area was obtained through eye-gaze, and then only few stimulus blocks began to flicker. Next, target classification was determined using EEG. Additionally, subjects could reject false triggering commands using eye closure. In this study, the system effectiveness was verified through offline experiment and online robotic-arm grasping experiment. Twenty subjects participated in offline experiment. For simultaneous mode, average ACC and ITR at the stimulus duration of 0.9 s were 90.50% and 60.02 bits/min, respectively. For sequential mode, average ACC and ITR at the stimulus duration of 1.4 s were 90.47% and 45.38 bits/min, respectively. Fifteen subjects successfully completed the online tasks of grabbing balls in both modes, and most subjects preferred the sequential mode. The proposed hybrid brain-computer interface (h-BCI) system could increase autonomy, reduce visual fatigue, meet individual needs, and improve the efficiency of the system.

## 1. Introduction

Brain-computer interface (BCI) is a communication system that transmits information between the brain and the outside world, without relying on the channels composed of muscles and nerves. Electroencephalography (EEG) is widely used for non-invasive BCIs because of its simplicity and safety (Sreeja and Samanta, 2019; Ke et al., 2020). Robotic arm control can help disabled people do rehabilitation training, such as motor function rehabilitation for stroke patients. In recent years, the use of EEG to control robotic arms has attracted researchers’ wide attentions, and has made great progress (Liu et al., 2018; Xu et al., 2019; Jeong et al., 2020; Zeng et al., 2020).

The BCI-controlled robotic arm systems can be divided into two types: synchronous systems and asynchronous systems (Diez et al., 2011). The synchronous systems operate according to a fixed time series. Chen et al. (2018) used the filter bank canonical correlation analysis (FBCCA) algorithm to implement a 7-DOF robotic arm control system based on steady-state visual evoked potentials (SSVEP) signals without system calibration. Later, Chen et al. (2019) proposed a new method combining high-frequency SSVEP with computer vision to complete the control of robotic arm. Ke et al. (2020) integrated augmented reality (AR) with BCI to provide a high-performance robotic arm control method. However, when the above-mentioned synchronous systems started, the users could not freely control systems’ start and stop. Thus, the asynchronous systems were proposed, in which a state switch was added to convert the states of working and idle. In this way, users could freely control the systems according to their wills. Pfurtscheller et al. (2010b) designed a brain switch based on motor imagery (MI) which was capable of activating an SSVEP-based four-step orthosis. Zhu et al. (2020) detected blinks using EOG to flicker the stimuli and completed the basic control of the 6-DOF robotic arm through SSVEP-BCI. Chen et al. (2021) used high-frequency SSVEP to switch the system state, and utilized low-frequency SSVEP to control the robotic arm to complete the puzzle tasks. In addition, MI was analyzed to change the command state autonomously to complete the multiple grasping tasks (Meng et al., 2016) and the continuous pursuit tasks (Edelman et al., 2019) of robotic arm.

In order to make good use of BCI for practical applications, more researchers pay attentions to hybrid brain-computer interfaces (h-BCIs) (Pfurtscheller et al., 2010a), especially those based on eye-tracking. Eye-tracking is characterized by easy acquisition and direct interaction. SSVEP is originally elicited by visual stimuli, which has high signal-to-noise ratio (SNR) and information transmission rate (ITR). Since simultaneous acquisition of the SSVEP and eye-tracking cannot bring additional burden to the subjects, the combination of the two modalities is natural for human-computer interaction (Kos’ Myna and Tarpin-Bernard, 2013). Stawicki et al. (2017) utilized eye-gaze to determine the stimulus range and then used SSVEP for fine-grained target selection. A hybrid system of spelling was realized, which enabled reliable control for most subjects. Lim et al. (2015) designed a three-region typing system. If the position of word selected by SSVEP was different from the region judged by eye-gaze, the current input would be rejected. Otherwise, the word would be inputted. Ma et al. (2018) realized a 40-target high-speed typing system integrating eye-gaze with SSVEP in VR environment. Mannan et al. (2020) combined eye tracking with SSVEP to achieve recognition of 48 targets using 6 stimulus frequencies, which improved the performance of the speller. Yao et al. (2018) reduced the number of SSVEP stimulus blocks by detecting eye-gaze before flickering stimulus, and realized a high-speed typing system in VR environment. A 30-target typing system was designed by Saboor et al. (2018). A single target was first selected by eye-gaze, and then the confirmation of the command was completed by high-frequency SSVEP. From these studies, two main modes of EEG-eye fusion could be summarized. The first one was to make fusion decision between EEG and eye-tracking (Lim et al., 2015; Ma et al., 2018; Mannan et al., 2020). The second one was to let eye-tracking and EEG execute sequentially to complete the task (Stawicki et al., 2017; Saboor et al., 2018; Yao et al., 2018). However, there were still some problems in the current researches on h-BCIs based on eye-tracking. First, many systems developed in the existing studies were still synchronous (Lim et al., 2015; Ma et al., 2018; Yao et al., 2018; Mannan et al., 2020), which had poor autonomy and flexibility. Second, most systems required a long time of calibration, such as the system calibration of the eye-tracking (Stawicki et al., 2017; Saboor et al., 2018; Mannan et al., 2020), and the training of the supervised algorithm (Ma et al., 2018; Yao et al., 2018; Tan et al., 2022), which were inconvenient for practical applications. Third, individual differences were not fully considered. In the BCI system, each subject had different performance in terms of stimulus interval, stimulus duration and susceptibility to fatigue. If system parameters were set without considering individual differences, system performance would be affected.

Aiming at the above problems and the two EEG-eye fusion modes, a robotic arm control system with simultaneous and sequential modes was implemented combining SSVEP with eye-tracking in virtual reality (VR) environment. For the simultaneous mode, the classification results were determined by the decision-level fusion of SSVEP and eye-gaze. In order to meet the individual needs of the subjects, the stimulus duration and fusion coefficient of each subject were determined by the adjustable window method in the calibration phase. In addition, subjects could control the start and stop of the system using triple blink and eye closure, so as to improve the subjects’ autonomy. For the sequential mode, no calibration was required before operation. In order to reduce subjects’ fatigues, the fixation area was determined by eye-gaze and then only half of the stimulus blocks flickered. And then the control command was selected using SSVEP. Subjects could switch the fixation area to freely control the start of the system. Moreover, subjects could close eyes during stimulus to avoid the execution of wrong commands caused by false triggers. In this study, offline and online experiments were conducted. The offline experiment was a cue-based stimulus experiment to demonstrate the effectiveness of the proposed system and determine parameters of the online system. In online experiment, subjects controlled the robotic arm to complete the ball-grabbing tasks in the two modes.

The main contributions and novelties of this study are as follows:

• In the VR environment, a high-performance virtual robotic arm control system combining EEG with eye-tracking was developed. It included simultaneous mode and sequential mode.
• For the simultaneous mode, the appropriate stimulus duration of each subject was determined by adjustable window method, which satisfied individual needs. And the start and stop of the system could be controlled by eye closure and triple blink, which improved subjects’ autonomous control ability.
• For the sequential mode, no calibration was required before use, which saved time and was conducive to practical application. And only half of the stimulus blocks flickered during the stimulus, which could reduce the fatigue of subjects. In addition, subjects were able to automatically control the start of the system by changing the fixation area.

The remainder of this study is organized as follows: Section “2. Materials and methods” introduces the structure of the proposed system, two control modes, data processing and experimental procedures. Section “3. Results” shows the results of offline and online experiments. Section “4. Discussion” discusses the performance of our proposed system and future work.

## 2. Materials and methods

### 2.1. System setup

#### 2.1.1. Architecture

**FIGURE 1:**
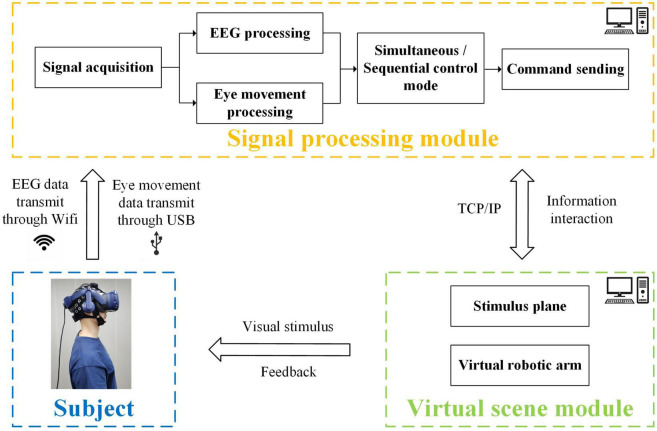
Schematic diagram of virtual robotic arm control system combining EEG and eye-gaze.

The structure of the robotic arm control system included virtual scene module and signal processing module, as shown in Figure 1. The system was built on a desktop computer with Intel Core i9 CPU and NVIDIA GTX3070 GPU. In virtual scene module, the construction of virtual scenes was realized by Unity 3D Engine and Steam VR platform. In signal processing module, the acquisition and real-time processing of EEG and eye-tracking data were completed. Real-time communication between both modules was completed through TCP/IP protocol. The head-mounted display HTC VIVE Pro was used to present the virtual scene, and its dual OLED display had a resolution of 2,880 × 1,600 (1,400 × 1,600 per eye) and a refresh rate of 90 Hz. An embedded infrared eye tracking module of aGlass DKII was used to acquire the eye movement data. A wireless 8-channel NeuSen W system equipment was utilized to collect EEG data. At the beginning of stimulus, in virtual scene module, eye movement indices were recorded and event triggers were sent to the parallel port to realize the simultaneous acquisition of EEG and eye movement data.

#### 2.1.2. Virtual scene

**FIGURE 2:**
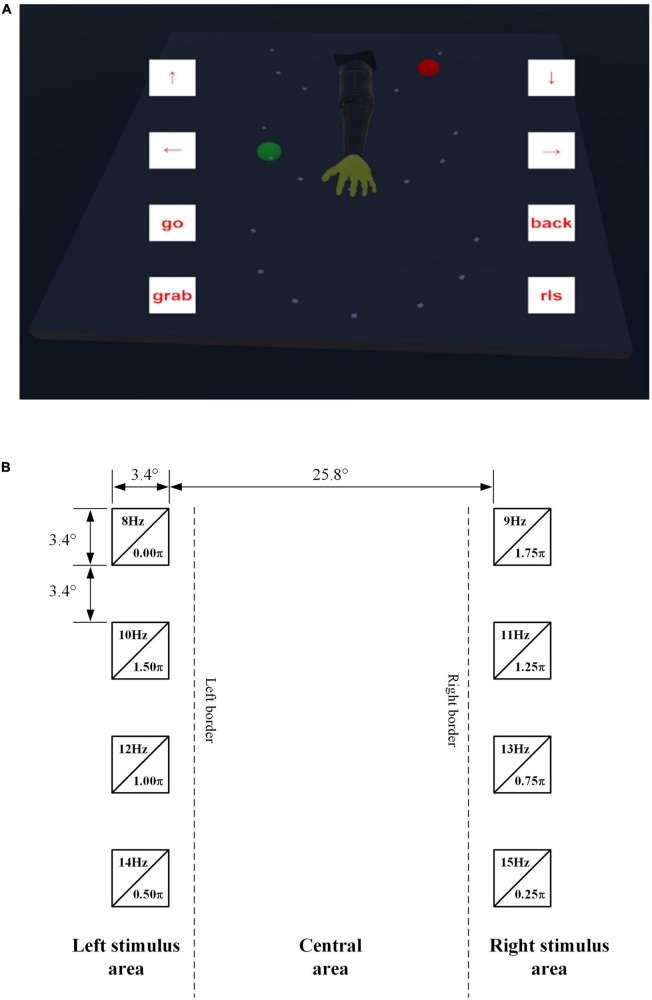
**(A)** Diagram of virtual scene. **(B)** Flickering stimulus blocks.

The virtual scene of this system mainly included two parts: the scene of robotic arm and the stimulus plane, as shown in Figure 2A. The scene of robotic arm contained a dark room, a table, a red ball and a robotic arm. There were a total of 21 light-colored markers with a green position on the table, which represented where the red ball might appear during the online experiment. The stimulus plane was a transparent canvas, which could move with subjects’ perspectives. The robotic arm could perform 8 commands. The 4 commands in the left stimulus area were “↑”, “←”, “go” and “grab”, which corresponded to the spinning up, turning left, going forward and grabbing actions of the robotic arm, respectively. The 4 commands in the right stimulus area were: “↓”, “→”, “back” and “rls”, corresponding to the spinning down, turning right, going back and releasing actions, respectively. Above 8 commands corresponded to 8 flickering stimulus blocks, as shown in Figure 2B. They were encoded in joint frequency-phase modulation (JFPM) mode (Chen et al., 2015b), whose frequency range was from 8 Hz to 15 Hz with 1 Hz interval, and whose phase range was from 0 to 2π with 0.25π interval. The entire fixation area was divided into three parts: central area, left stimulus area and right stimulus area. Both length and width of each flickering stimulus block were 3.4°, where ° was the unit of viewing angle. The vertical distance between two flickering stimulus blocks on the ipsilateral side was 3.4°, and the horizontal distance between the left and right flickering stimulus blocks was 25.8°.

### 2.2. Online control mode

**FIGURE 3:**
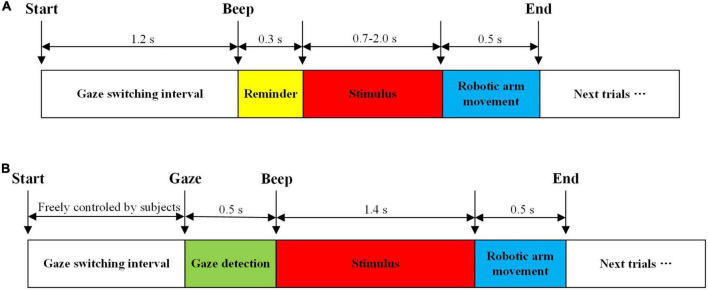
Implementation procedure. **(A)** Simultaneous mode. **(B)** Sequential mode.

This study utilized the advantages of EEG and eye movement to develop a convenient and high-performance h-BCI system. Based on the combination of EEG and eye movement, two online control modes were proposed in our study: simultaneous mode and sequential mode, as shown in Figure 3.

#### 2.2.1. Simultaneous mode

System calibration was needed before operation. In this mode, eye-gaze was involved in the fine classification of targets, so five-point calibration of eye-tracking equipment was required. In addition, EEG-eye fusion required calibration to determine the fusion coefficients. First, five-point calibration of eye-gaze was conducted. Second, subjects were required to successively gaze at each stimulus block for 2 s to calculate the gaze-center coordinates of 8 stimulus blocks. Third, subjects stared at 8 stimulus blocks in turn again, and each stimulus block flickered for 2 s. Meanwhile, the eye movements and EEG data were collected synchronously. The adjustable window method was used to change the time window to calculate the classification accuracy of EEG and eye movements, so as to determine the optimal stimulus duration and fusion coefficient for each subject.

The adjustable window method was to intercept EEG and eye-gaze data in the range of 0.7-2 s with a step of 0.1 s. The current period was assumed as *T*. First, for three consecutive periods of *T* − 0.1, *T*, and *T* + 0.1, their classification accuracies of the fusion between EEG and eye-gaze needed to be all greater than or equal to 87.5%. Next, if the classification accuracy in the period of *T* was greater than 87.5%, *T* was selected as the stimulus duration. If the classification accuracy in the period of *T* was equal to 87.5%, and the output labels in the periods of *T* and *T* − 0.1 were different, *T* was selected as the stimulus duration. Otherwise, the time period would be increased till the period *T* satisfied the above conditions, and then the stimulus duration was found. If *T* satisfying the precision condition was not found, the stimulus duration would be set as 2 s. Because of the less calibration data, when the stimulus duration was short, the classification accuracy might be unstable, or some commands could not be recognized. Considering the situations, the adjustable window method was proposed to ensure that subjects could successfully complete the online experiment.

The implementation procedure of the simultaneous mode is shown in Figure 3A. First, the gaze switching interval was set at 1.2 s, in which subjects could switch the gaze to find the target. Afterwards, there was a beep to remind subjects to focus on the target stimulus block. After beeping for 0.3 s, stimulus blocks began to flicker, and the stimulus duration of flickers was determined by the adjustable window method for each subject. And then EEG and eye-gaze data with a length of a stimulus duration were intercepted. The classification result was determined by the decision-level fusion of EEG and eye-gaze. The robotic arm then performed relevant action, and the corresponding stimulus block turned blue. In addition, there was a voice prompt to provide feedback to subjects, which was the Chinese pronunciation of the command for the corresponding stimulus block. Later, the system entered the next trial, and automatically repeated the above procedure until the end of the task.

When subjects felt fatigued or were unable to keep up with the control rhythm, they could close their eyes during stimulus to stop the system. After that, subjects could think about the next control command or have a rest. Meanwhile, a sliding window with a length of 3 s and a step of 0.1 s started to detect subjects’ triple blink. If subjects wanted to restart the system, they could blink three times in a row.

#### 2.2.2. Sequential mode

No system calibration was required before operation. In this mode, eye-gaze was only utilized to roughly estimate the location of stimulus blocks, and then the final target was classified by EEG. Therefore, eye-gaze could require neither the calibration of eye-tracking equipment nor the calibration to determine the EEG-eye fusion coefficients. The implementation procedure of the sequential mode is shown in Figure 3B. In the gaze switching interval, subjects utilized eye-gaze to freely select the target. During this period, a sliding window with a length of 0.5 s and a step of 0.1 s was used to detect subjects’ gazes. If the variance of eye-gaze was smaller than the predetermined threshold (≤ 0.005), it was judged as gaze state. In the gaze state, if gaze center fell on the area to the left of the left boundary or the area to right of the right boundary (i.e., left or right stimulus areas in Figure 2B), the system beeped and partial stimulation started, that is, the four ipsilateral stimulus blocks started to flicker simultaneously for 1.4 s. Then, the control command was selected using SSVEP. The execution process of the robotic arm and the feedback to the subjects in this mode were the same as those in the simultaneous mode. The system repeated the above procedure until the end of the task.

In this mode, subjects could use eye-gaze to switch the fixation area, so as to freely control the start of the system. In addition, due to subjects’ sight shift, false triggers might occur, which caused the system to send wrong commands. At this time, subjects could close their eyes during stimulus, and the robotic arm could refuse to execute the wrong commands.

### 2.3. Data processing

#### 2.3.1. EEG

First, a bidirectional zero-phase Chebyshev type I IIR filter was used for 7-90 Hz bandpass filtering (Ke et al., 2020), and a 50 Hz digital notch filter was used to filter out power frequency interference. Next, baseline removal of each trial was performed using EEG data pre-trigger 200 ms. Finally, considering the visual latency of 0.14 s (Chen et al., 2015b), the effective EEG signal was intercepted in [0.14 s 0.14+x s], where x was the stimulus duration.

Considering the contribution of high-order harmonics with high SNR in SSVEP data to target classification, the FBCCA (Chen et al., 2015a) algorithm was used to classify SSVEP data in this study. This algorithm first used a filter bank to decompose the preprocessed EEG data into multiple sub-band components. Then according to the principle of CCA algorithm (Lin et al., 2006), the correlation coefficients of all sub-bands were obtained. Finally, a weighted method combining these coefficients was utilized to obtain the feature for target classification, and the frequency corresponding to the maximum feature was selected as the target.

#### 2.3.2. Eye-tracking

For the simultaneous mode, the gaze-center coordinates of the 8 targets needed to be obtained in the calibration phase. First, the zero points of the binocular eye-gaze data were removed, which might be caused by subjects’ eye closure or the loss of sampling data. Second, the average values of all sampling points of left and right eye-gaze data were calculated, respectively. And then the gaze-center coordinates of 8 targets were obtained. In the phase of target recognition, the sum of the Euclidean distances between the test data and 8 gaze-center coordinates were calculated point by point. Finally, the target with the smallest sum of Euclidean distances was selected.

**FIGURE 4:**
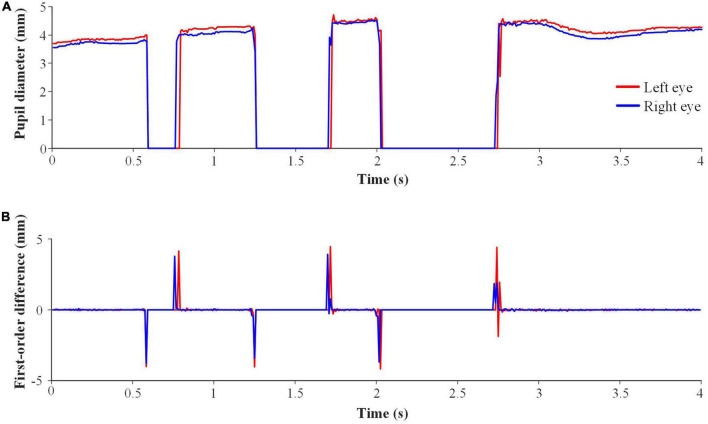
Schematic diagram of triple blink data. **(A)** Original pupil diameter. **(B)** First-order difference.

Triple blink was detected to restart the system. The general binocular pupil diameters of triple blink are shown in Figure 4A. There are three segments with pupil diameters of 0, which correspond to three eye closures in triple blink. The first-order difference was used to detect the triple blink, as illustrated in Figure 4B. The brief steps of triple blink detection in a sliding window were as follows. First, the first 8 positive peaks and their time points were found. The number of peaks was set to 8 according to prior knowledge. Second, if the intervals of these time points were within 1/12 s, their amplitudes would be summed. Third, the first 8 negative peaks were dealt with in the same way. Fourth, if the intervals of the time points between positive and negative peaks were within 1/24 s, the peak with smaller absolute value would be deleted. Fifth, if the absolute amplitudes of the first 3 positive and 3 negative peaks among the remaining ones were all greater than 2.5 mm, then triple blink for one eye was detected. Finally, if at least one triple blink was detected for both eyes, it was judged as triple blink in the sliding window.

Eye closure was detected to pause the system. If the non-zero values of the binocular pupil diameters accounted for less than 30% in the whole stimulus duration, it was judged as eye closure in the stimulus duration.

For the sequential mode, the gaze state needed to be detected. First the zero points of the binocular fixation coordinates were removed, and then the variance of binocular eye-gaze data during fixation was calculated. If the variance was less than a predetermined threshold, it was indicated that gaze state was detected. In addition, eye-closure detection was used to refuse the false triggers, and the detection method was the same as that in the simultaneous mode.

#### 2.3.3. EEG-eye fusion method

The decision-level fusion method between EEG and eye-gaze proposed in this study is as follows:


$$\rho_{f\,u\,s\,e}=C_{e\,y\,e}\times W_{e\,y\,e}\times N\,o\,r\,m\,\left(\frac{1}{\rho_{e\,y\,e}}\right)+W_{e\,e\,g}\times N\,o\,r\,m\,\left(\rho_{e\,e\,g}\right),$$

Where ρ_*fuse*_ is the final fusion coefficient. Since the eye movement data may be lost during acquisition, the eye-tracking credibility *C*_*eye*_ is utilized. If there are no valid data for both eyes, *C*_*eye*_ = 0, otherwise *C*_*eye*_ = 1. *W*_*eye*_ and *W*_*eeg*_ are the fusion weights of eye-gaze and EEG data, respectively. *Norm*(⋅) is a linear normalization function. The normalized value can be regarded as the output probability of each target, and ∑*Norm*(⋅) = 1. ρ_*eye*_ is the eye-gaze coefficient obtained through the Euclidean distance method. Since the target with the minimum value of the eye-gaze coefficient is selected, the reciprocal of the eye-gaze coefficient is conducted. ρ_*eeg*_ is the EEG coefficient obtained using FBCCA algorithm. Finally, the target with the largest fusion coefficient ρ_*fuse*_ is desired.

The performance of three fusion methods including average fusion, prior fusion, and particle swarm optimization (PSO) fusion were compared in this study. The weights of eye-gaze and EEG in the average fusion were the same, that is, *W*_*eye*_ = *W*_*eeg*_ = 0.5. The weights of eye-gaze and EEG in the prior fusion were determined by the classification accuracy in calibration phase (Ma et al., 2018), that is, *W*_*eye*_ = (*ACC*_*eye*_)^2^, *W*_*eeg*_ = (*ACC*_*eeg*_)^2^. For the PSO fusion, the particle swarm optimization algorithm was used to obtain the weights of eye-gaze and EEG (Tan et al., 2022).

### 2.4. Subjects

Twenty-four healthy subjects (14 males and 10 females, aged from 22 to 26 years old) participated in the experiments, all of whom had normal or corrected-to-normal visions. 20 subjects participated in the offline experiment, and 15 subjects participated in the online experiment, of whom 11 subjects participated in the both. All subjects signed the consent form before the experiment. The experiments complied with the Declaration of Helsinki and was approved by the local ethics committee. Wearing both a head-mounted display and an EEG cap, subjects sat comfortably in a sound-proof room to complete all the experiments.

### 2.5. Data acquisition

For EEG, according to the International 10-20 system, the EEG amplifier collected 8-channel EEG data (PO5, PO3, POZ, PO4, PO6, O1, OZ, O2), and Cz and AFz were reference and ground electrodes, respectively. The sampling rate was set to 1000 Hz. The impedance of each channel was kept less than 10 kΩ.

For eye-tracking, the eye-gaze data and pupil diameters of left and right eyes were collected.

### 2.6. Experiment design

Offline and online experiments were carried out in this study.

#### 2.6.1. Offline experiment

**FIGURE 5:**
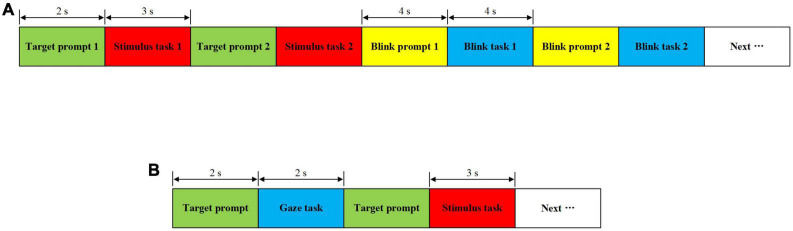
Schematic diagram of the offline experiment procedure. **(A)** Offline simultaneous mode. **(B)** Offline sequential mode.

In order to verify the validity of the stimulus paradigm designed in the 3D scene and determine the relevant parameters of the online system, the offline experiment was conducted, including offline simultaneous and sequential modes. Each mode had two sessions, and each session contained six blocks. Offline simultaneous mode comprised Session 1 and 2, whereas offline sequential mode comprised Session 3 and 4. These two experiment procedures are shown in Figures 5A, B, respectively.

For the experiment procedure of the offline simultaneous mode, there were two stimulus tasks. For each stimulus task, a visual cue lasted for 2 s to remind subjects to gaze the target which was random in the eight stimulus blocks. Then, all the stimulus blocks flickered simultaneously for 3 s. Next, there were two blink tasks. For each blink task, a blink prompt lasted for 4 s, and then subjects were asked to perform the corresponding blinking action within 4 s. The above process was repeated four times to form a block. In addition, for each session, the eye closure and triple blink tasks were interspersed in Block 1, 3, and 5, meanwhile, single and double blink tasks were interspersed in Block 2, 4, and 6.

For the experiment procedure of the offline sequential mode, a visual cue first lasted for 2 s, and then subjects were asked to gaze at the target block for 2 s. Next, the same target cue was kept for 2 s, and then the four stimulus blocks in the same stimulus area as the prompted target flickered simultaneously for 3 s. The above process was repeated 8 times for a block.

In order to improve subjects’ attentions, there were beeps at the start and end of all stimulus tasks, blink tasks and gaze tasks. During the visual cue, the target would be highlighted as a green square, and subjects could shift their gaze to the target. After a block, subjects had a rest for 10 s. After one session, subjects took off the head-mounted display and rested for 5 min. In addition, a five-point calibration was required for Session 1 and 3, but not for Session 2 and 4 in which the same configuration was used. For stimulus tasks and blink tasks, EEG and eye movement data were collected simultaneously. For gaze tasks, only eye-movement data were collected.

#### 2.6.2. Online experiment

In online experiment, subjects were asked to control the virtual robotic arm to complete 5 tasks of grabbing balls in two control modes. As shown in Figure 2A, the goal of each grasping task was to grab the red ball and put it in the green position.

For each grasping task, subjects first needed to plan the moving path of the robotic arm, and used “go”, “back”, “←” and “→” commands to control the robotic arm to move over the red ball, and then utilized the “↓” command to approach the ball. When the robotic arm moved in the horizontal plane by executing the commands “go”, “back”, “←” and “→”, it would move one grid between the adjacent light-colored marks each time. And the robotic arm executed “↓” once to get ready to grab the ball. After that, “grab” command was used to grab the ball. Next, “↑” command was needed to restore the horizontal position, then the red ball was moved over the green position, and finally “↓” and “rls” commands were performed in sequence to complete the task. If the robotic arm moved incorrectly, subjects should re-plan the path to complete the grasping task. Finally, if the position of the red ball coincided with the green position, it was indicated that the ball was correctly placed and the current grasping task was successful. If subjects completed 5 grasping tasks without mistakes, the total steps were 72.

There were some rules during the online experiment. For example, “grab” action could not be executed twice in a row, neither could “rls” action. After the robotic arm performed “↓” command, the robotic arm could not execute “go”, “back”, “←” or “→” commands. If the above situations occurred, the system would prompt subjects “this command is invalid, please resend the command”.

Before the formal experiment, subjects completed a pre-experiment of grabbing balls to ensure that they were familiar with the two control modes. In the formal experiment, the simultaneous and sequential modes were performed randomly for each subject. In addition, a stopwatch was used to record the experimental time. After the formal experiment, each subject was required to complete a questionnaire about the performance of the two control modes, and then subjective evaluation results were obtained.

### 2.7. Evaluation metrics

SNR was used to evaluate the quality of the elicited SSVEP signals in VR environment (Wang et al., 2017; Yao et al., 2019). SNR here was the ratio of the amplitude at the target frequency to its average amplitude within 2 Hz of its vicinity.

In offline experiment analysis, the performance evaluation metrics of the system were classification accuracy (ACC) and ITR. ITR in bits/min is a widely used criterion to evaluate the BCI performance (Wolpaw et al., 2002; Chen et al., 2020):


$$I\,T\,R=\frac{60}{T}\times \left(l\,o\,g_{2}\,M+P\,l\,o\,g_{2}\,P+\left(1-P\right)\,l\,o\,g_{2}\,\left(\frac{1-P}{M-1}\right)\right),$$

Where *M* is the number of targets (*M* = 8 in this study), *P* is ACC, and *T* is the time required to send a command.

In online experiment analysis, the evaluation metrics were the time and steps required to complete the grasping tasks, and the questionnaires based on 5-point Likert scale. The approval level ranged from 1-5, where 1 indicated the strongest disagreement and 5 indicated the strongest agreement.

## 3. Results

### 3.1. Offline experiment

#### 3.1.1. SNR of SSVEPs in VR environment

**FIGURE 6:**
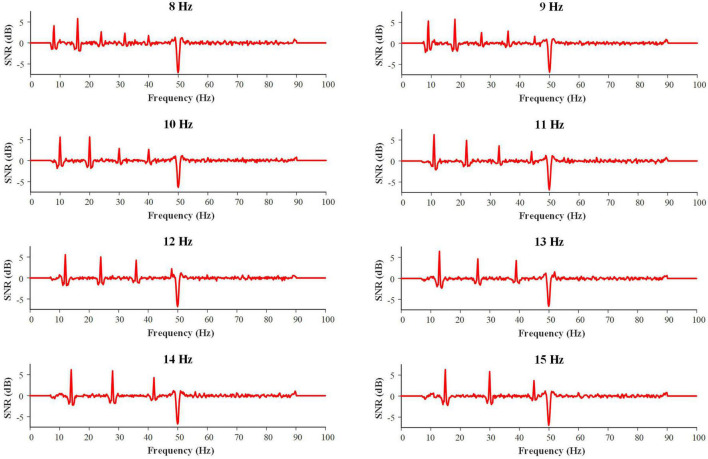
SNR curves of SSVEPs at all 8 stimulus frequencies.

Figure 6 shows the grand average SNR curves of the SSVEPs for all 8 stimulus frequencies. It was found that the SNR curves had obvious peaks at the fundamental frequency and co-frequency of the target frequency, and the obvious sagged at 50 Hz which was the result of a notch filter. The results illustrated the effectiveness and stability of stimuli in VR environment.

#### 3.1.2. Comparison of fusion methods between EEG and eye-gaze in offline simultaneous mode

**FIGURE 7:**
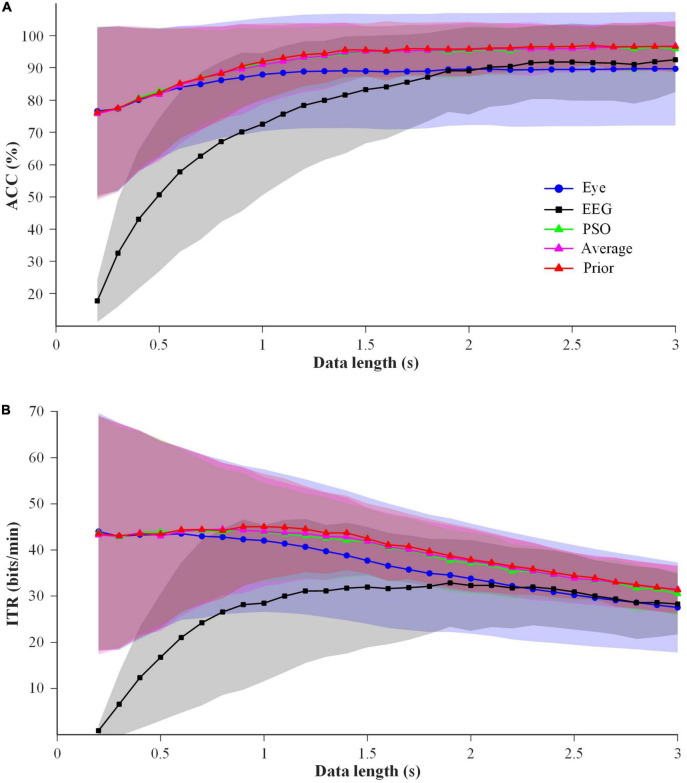
The performance comparison of the EEG-eye fusion methods with 1 block data for training and 1 block data for testing at data length of 0.2–3 s for all subjects. Shaded area represents standard deviation **(A)** ACC. **(B)** ITR.

In order to select a superior EEG-eye fusion method for online simultaneous mode, the performance of average fusion, prior fusion and PSO fusion using the data of 6 blocks in Session 1 was analyzed. Considering the short training time required for online applications, the process of the former one block for training and the latter one block for testing was sequentially conducted 6 times for 6 blocks. The results of ACC and ITR are shown in Figure 7. Two-way repeated-measure ANOVAs (method × time) were performed on ACC and ITR, respectively. The results showed that two factors had significant main effects on ACC and ITR: method (ACC: *F*(4,76) = 20.731, *p-value* < 0.01; ITR: *F*(4,76) = 29.229, *p-value* < 0.01) and time (ACC: *F*(28,532) = 34.619, *p-value* < 0.01; ITR: *F*(28,532) = 11.369, *p-value* < 0.01). Significant interaction effect between method and time was found (ACC: *F*(112,2128) = 40.076, *p-value* < 0.01; ITR: *F*(112,2128) = 34.241, *p-value* < 0.01). And the pairwise comparisons for “method” using LSD test showed that ACCs of all three fusion methods were significantly higher than those of the single-modal EEG data (*p-values* < 0.01) and single-modal eye-gaze data (*p-values* < 0.05), meanwhile the ITRs of both average fusion and prior fusion were significantly better than those of single-modal EEG data (*p-values* < 0.01) and single-modal eye-gaze data (*p-values* < 0.05). And the ACC and ITR of prior fusion were significantly better than that of the other two fusion methods (*p-values* < 0.05), whereas there was no significant difference between average fusion and PSO fusion (*p-value* > 0.05). In addition, one-way repeated-measure ANOVAs (method) were performed at each data length, and it was found that the ACCs and ITRs of the prior fusion were significantly higher than those of single-modal eye-gaze data after 0.7 s (except 0.8 s) (*p-values* < 0.05) and those of single-modal EEG data at all data lengths (*p-values* < 0.05). In general, the performance of prior fusion was the best, so it was selected as the EEG-eye fusion method of online simultaneous mode.

#### 3.1.3. Blink detection

To validate the effectiveness of the proposed eye closure and triple blink detection methods, ACC, true positive rate (TPR) and false positive rate (FPR) were calculated using the eye movement data of eye closure, single blink, double blink, and triple blink. The average ACC, TPR and FPR of eye closure detection were 99.64%, 98.96% and 0.14%, respectively. Meanwhile, those of triple blink detection were 95.83%, 88.95% and 1.88%, respectively. Therefore, eye closure and triple blink could be used in the online system to accomplish the corresponding controls.

#### 3.1.4. Detection of left and right stimulus areas in offline sequential mode

**FIGURE 8:**
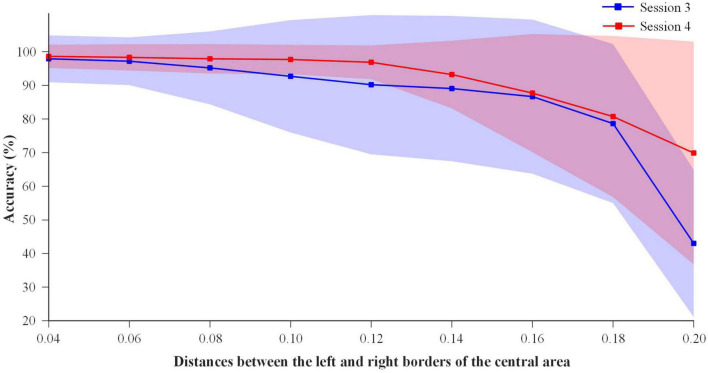
Detection accuracy of the left and right stimulus areas for sequential mode in the offline experiment.

In order to divide the left and right stimulus areas in VR environment for the online sequential mode, the eye movement data of the gaze tasks in Session 3 (with calibration) and Session 4 (without calibration) were analyzed to determine the stimulus area where subjects’ fixation points were located. As shown in Figure 2, the area to the left of the central area was regarded as the left stimulus area, and the area to the right of the central area was regarded as the right stimulus area. Figure 8 shows the detection accuracy of the stimulus areas at different distances between the left and right borders of the central area. As the increase of the distance, the detection accuracy of the stimulus areas decreased for Session 3 and 4. Paired T-tests were performed on the detection accuracy of Session 3 and 4 at the distances of 0.04-0.20. When the distance was 0.20, the accuracy of Session 4 was significantly higher than that of Session 3 (*p-value* < 0.01), and the statistical results had no significant differences at the other distances (*p-values* > 0.05). It was indicated that whether or not the five-point calibration of eye tracker was performed made little difference in the detection of the stimulus area at the distances of 0.04-0.18.

For the online sequential mode without five-point calibration, if the distance was smaller, the detection accuracy would be higher, but there would be less free fixation area left for subjects and more false triggers. And if the distance was larger, the detection accuracy would be lower. Taking the above factors into consideration, the distance between the left and right borders of the central area was set at 0.12, so as to ensure that subjects can freely and accurately select the stimulus areas through eye tracking.

#### 3.1.5. Comparison of EEG classification performance in offline simultaneous and sequential modes

**FIGURE 9:**
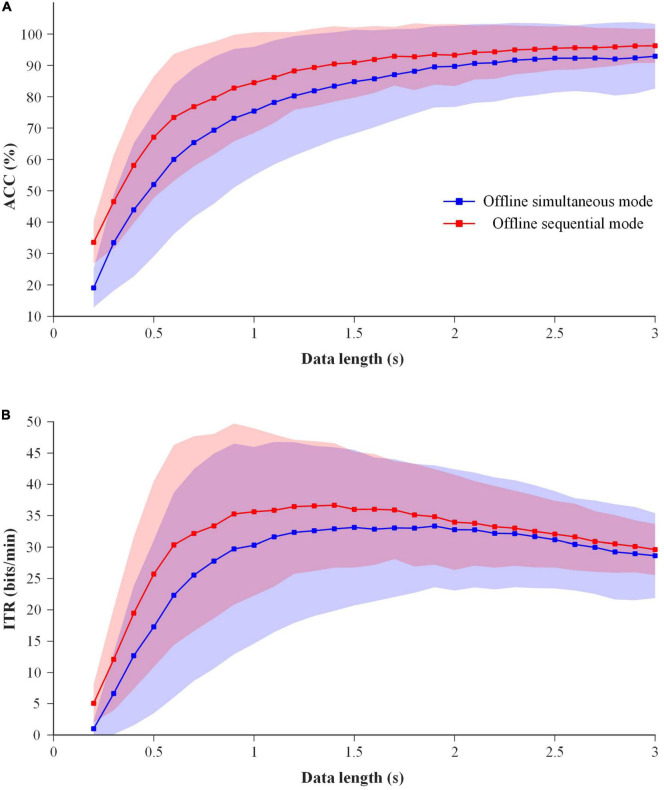
Comparison of EEG classification performance between offline simultaneous and sequential modes at data length of 0.2–3 s. **(A)** ACC. **(B)** ITR.

In order to determine the suitable stimulus duration of EEG for online sequential mode, the EEG data of both offline simultaneous and sequential modes were analyzed. Figure 9 shows the classification ACCs and ITRs of EEG data at data length of 0.2–3 s in two offline modes with a time step of 0.1 s. To calculate ITR in the offline experiment, the gaze switching interval of each mode was 2 s. Two-way repeated-measure ANOVAs (mode × time) were performed for ACC and ITR. The results showed that two factors had significant main effects on ACC and ITR: mode (ACC: *F*(1,19) = 23.519, *p-value* < 0.01; ITR: *F*(1,19) = 11.994, *p-value* < 0.01) and time (ACC: *F*(28,532) = 143.593, *p-value* < 0.01; ITR: *F*(28,532) = 49.813, *p-value* < 0.01). Significant interaction effect between mode and time was found (ACC: *F*(28,532) = 16.217, *p-value* < 0.01; ITR: *F*(28,532) = 10.049, *p-value* < 0.01). And the pairwise comparisons for “method” using LSD test showed that the ACCs and ITRs of EEG data in offline sequential mode were significantly higher than those in offline simultaneous mode (*p-values* < 0.01). It was indicated that partial stimulation in the sequential mode could increase the classification performance.

Figure 9 shows that the ACC of EEG data in offline sequential mode exceeds 90% for the first time at the stimulus duration of 1.4 s, and the ITR at this stimulus duration is the highest. In order to ensure that most subjects can successfully complete the grasping tasks, the stimulus duration of EEG in online sequential mode was set to 1.4 s for all subjects.

### 3.2. Online experiment

#### 3.2.1. Performance comparison of simultaneous and sequential modes

In online experiment, all 15 subjects successfully completed the tasks of grabbing balls with two control modes. Table 1 shows the performance comparison between simultaneous and sequential modes. For the simultaneous mode, the averages of completion time, calibration time and steps were 356.47 s, 126.47 s and 86.67, respectively. For the sequential mode, the averages of completion time and steps were 347.33 s and 84.73, respectively. For the simultaneous mode, the stimulus duration ranged from 0.7 to 1.7 s (mean, 0.89 s), indicating that the proposed adjustable window method was effective. And all subjects used 0-2 pauses (mean, 0.87), demonstrating that the proposed state switch was necessary and effective. For the sequential mode, only a few subjects conducted the eye closure operation, illustrating that the false triggering of the system existed but was rare, and it was necessary and effective to reject false triggers using eye closure.

**TABLE 1:** Performance comparison of each subject between two modes in online experiment.

Subjects	Simultaneous mode					Sequential mode			Preferred mode
	**Completion time (s)**	**Calibration time (s)**	**Stimulus duration (s)**	**Steps**	**Pause times**	**Completion time (s)**	**Steps**	**Refusal times**	
1	391	124	0.7	96	2	316	81	0	Sequential
2	359	125	0.7	85	2	310	80	0	Sequential
3*	420	137	1.7	86	0	314	87	0	Sequential
4	276	129	0.7	72	0	379	98	0	Simultaneous
5	336	130	1.0	82	0	304	79	0	Sequential
6*	366	124	0.8	87	2	377	85	9	No preference
7*	366	124	0.9	81	2	335	81	0	No preference
8	341	132	0.7	92	0	335	79	0	Sequential
9	320	124	0.7	82	1	320	83	0	No preference
10	339	124	1.2	79	0	289	75	1	Sequential
11	320	124	0.7	78	1	314	72	0	Sequential
12*	395	124	1.2	87	2	458	100	1	Simultaneous
13	340	125	1.0	84	0	341	83	0	No preference
14	403	124	0.7	106	1	395	88	1	Sequential
15	375	127	0.7	103	0	423	100	5	No preference
Mean ± std	356.47 ± 37.59	126.47 ± 3.91	0.89 ± 0.28	86.67 ± 9.23	0.87 ± 0.88	347.33 ± 48.62	84.73 ± 8.61	1.13 ± 2.45	

*Represents new subjects who did not participate in offline experiment.

Paired *T*-test was used to compare the same metrics between the two modes, and the results showed that no significant differences between the two modes were found for completion time and steps (*p-values* > 0.05), respectively. However, if the completion time of simultaneous mode included calibration time, the completion time of sequential mode was significantly lower than that of simultaneous mode (*p-value* < 0.01).

Taking into account completion time and steps, each subject’s mode preference was analyzed. Among them, Subject 4 and 12 preferred the simultaneous mode. Subject 1, 2, 3, 5, 8, 10, 11, and 14 preferred the sequential mode. And the remaining subjects had no obvious preference.

#### 3.2.2. Subjective evaluation of simultaneous and sequential modes

After the online experiment, subjects were required to complete a questionnaire. According to 5-point Likert scale, the controllability, comfort, friendliness and other aspects of the two modes were compared in the questionnaire. The subjective evaluation results of the questionnaire are shown in Table 2. There were no significant differences in the scores of five questions between simultaneous and sequential modes (*p-values* > 0.05), respectively. However, the overall average scores of the sequential mode were higher than those of the simultaneous mode. Moreover, the average scores of all questions in the two modes exceeded 4 except for the third question, indicating that the two modes had great performance in stable controllability, less fatigue, high accuracy, and good friendliness.

**TABLE 2:** Subjective evaluation results of each subject on two modes.

Subject	The system can be controlled easily		My eyes do not get tired easily		I do not need to excessively focus my attention		Commands are always recognized according to my wishes		This control mode is very friendly	
	**Simultaneous mode**	**Sequential mode**	**Simultaneous mode**	**Sequential mode**	**Simultaneous mode**	**Sequential mode**	**Simultaneous mode**	**Sequential** **mode**	**Simultaneous mode**	**Sequential mode**
1	4	5	5	5	4	4	4	5	4	4
2	4	5	4	4	4	4	5	4	4	5
3*	2	3	3	4	2	2	4	4	4	5
4	5	4	5	5	5	5	5	4	5	4
5	5	4	4	4	5	4	4	4	5	4
6*	4	4	3	3	3	3	3	3	3	3
7*	4	3	3	4	4	3	4	4	4	4
8	3	4	4	4	4	4	3	4	3	4
9	5	4	5	5	5	5	5	4	5	4
10	2	3	4	4	2	2	3	4	4	4
11	4	5	4	4	3	3	5	5	5	5
12*	5	4	5	4	3	3	4	4	5	5
13	4	5	5	5	4	5	4	4	4	5
14	5	4	5	5	4	4	4	5	5	5
15	5	5	5	5	4	3	4	4	3	4
Mean ± std	4.07 ± 1.00	4.13 ± 0.72	4.27 ± 0.77	4.33 ± 0.60	3.73 ± 0.93	3.60 ± 0.95	4.07 ± 0.68	4.13 ± 0.50	4.20 ± 0.75	4.33 ± 0.60

*Represents new subjects who did not participate in offline experiment.

## 4. Discussion

In this study, a virtual robotic arm control system combining EEG and eye-tracking in VR environment was developed, and two control modes including simultaneous and sequential modes were proposed.

### 4.1. Performance comparison of robotic arm control systems with previous studies

In order to illustrate the advantages of the proposed robotic arm control system, the performance of the two control modes in this study were compared with that of the existing synchronous and asynchronous robotic control systems. The results are shown in Table 3.

**TABLE 3:** Performance comparison of robotic arm control systems among different studies.

Control mode	References	Offline ACC (%)	Offline ITR (bits/min)	Online effective commands	Online completion time (s)	Average time per command (s)	Adjustable stimulus duration	System calibration	Partial stimulation	Start-stop way	Command confirmation	False trigger rejection
Synchronous	Chen et al., 2018	92.78	49.25	159.83	639.33	4.00	No	No	No	No	No	No
	Chen et al., 2020	94.06	14.41	15.83	107.67	6.80	No	No	No	No	No	No
	Chen et al., 2019	97.75	17.00	16.00	104.00	6.50	No	No	No	No	No	No
Asynchronous	Gao et al., 2017	93.00	18.43	16.25	297.37	18.30	No	Yes	No	MI	No	Yes
	Zhu et al., 2020	92.09	35.98	68.73	387.33	5.64	No	Yes	No	EOG	Yes	No
	Chen et al., 2021	94.97	67.37	19.40	701.70	36.17	Yes	Yes	No	SSVEP	Yes	No
	Proposed simultaneous mode	90.50	60.02	86.67	356.47	3.77	Yes	Yes	No	Eye-tracking	No	No
	Proposed sequential mode	90.47	45.38	84.73	347.33	3.75	No	No	Yes	Eye-tracking	No	Yes

For the proposed simultaneous mode, since the average stimulus duration of the online experiment was 0.89 s, the offline ACC and ITR were calculated at this stimulus duration. For the proposed sequential mode, considering that the average gaze switching interval of the online experiment was 1.25 s, the offline ITR was calculated according to the average gaze switching interval. Although the offline ACCs of the two proposed modes were not the highest, but the offline ITRs were higher than most of other systems, because the output time of a command was shorter than that of other studies. For the study (Chen et al., 2021), four classifications were required for one effective online command, but the offline ITR was calculated using the output time of one classification.

For the online tasks, the average completion time per command of the proposed simultaneous and sequential modes reached 3.77 s and 3.75 s, respectively, which outperformed other systems.

As for system start-stop ways of asynchronous systems, MI, EOG, SSVEP, and eye-tracking were utilized in the studies (Gao et al., 2017; Zhu et al., 2020; Chen et al., 2021) and this study, respectively. MI detection in study (Gao et al., 2017) required a long time about 4 s for each trial and average detection accuracy was about 75%. For the study (Zhu et al., 2020), individual calibration of EOG was needed before online operation, and it took 3 s to complete one start or stop for several trials. And SSVEP used in the study (Chen et al., 2021) required continuous flickering stimulus about 2.8 s for each trial, which might increase subjects’ fatigue. Eye-tracking used in the two proposed modes was more natural and efficient for interaction. The proposed simultaneous mode needed 3 s to start and one stimulus duration (0.7-1.7 s) to stop for several trials, and the proposed sequential mode needed only 0.5 s to start for each trial. Therefore, start-stop way of the proposed system saved more time than other systems.

For additional system functions, although command confirmation for each trial in the studies (Zhu et al., 2020; Chen et al., 2021) could make the system more stable, it came at a cost of runtime. Teeth clenching was utilized in the study (Gao et al., 2017) to realize the false-trigger rejection, whereas eye closure was used in this study. Adjustable stimulus duration of single EEG modality was conducted for each trial in the study (Chen et al., 2021), whereas adjustable stimulus duration was determined by the fusion result of EEG and eye-gaze for each subject in this study. Furthermore, the proposed sequential mode also featured no calibration, but other asynchronous system all needed calibration. Compared with other previous studies, partial stimulation was only utilized in the proposed sequential mode.

Furthermore, some studies utilized MI to freely control robotic arms and effectively completed grasping tasks (Meng et al., 2016) and the continuous pursuit tasks (Edelman et al., 2019). But in the two studies, the training time required was long. The number of commands was only 4, and a larger number of electrodes were used, that is, 64 in Meng et al. (2016) and 57 in Edelman et al. (2019). In the proposed system, simultaneous mode and sequential mode improved on the above problems. Only two runs of calibration were required for simultaneous mode, and no calibration was needed for sequential mode. The number of commands was set to 8 according to the grasping task, and there was still room for expansion. The number of electrodes used in the proposed online system was 8, which was good for practical applications. In addition, two modalities of EEG and eye-tracking were utilized to improve the system performance.

The above results indicated that the proposed system had high performance of human-computer interaction, such as great autonomy, good interaction-friendliness, and high efficiency.

### 4.2. Fusion method of EEG and eye-gaze

**FIGURE 10:**
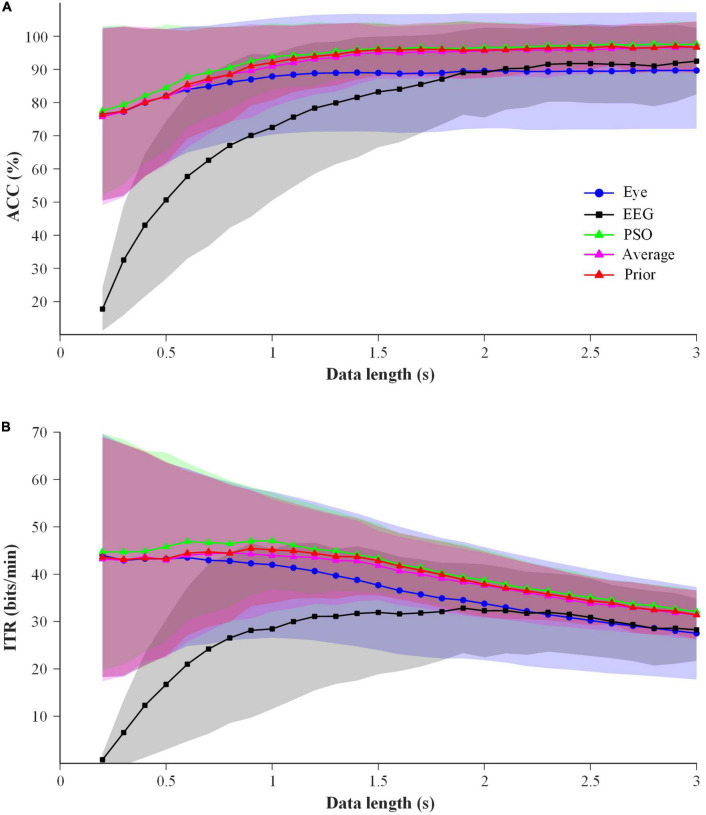
The performance comparison of the EEG-eye fusion methods with 5 block data for training and 1 block data for testing at data length of 0.2–3 s for all subjects. Shaded area represents standard deviation **(A)** ACC. **(B)** ITR.

In this study, the performance of prior fusion was better than average fusion and PSO fusion under the condition of 1 block for training and 1 block for testing. However, in the previous study (Tan et al., 2022), the performance of PSO was optimal. The reason was explained as follows. The performance of several fusion methods was compared using 5 blocks of data for training and 1 block for testing. The results are shown in Figure 10. Two-way repeated-measure ANOVAs (algorithm × time) were performed on ACC and ITR, and the results showed that the performance of the PSO fusion method was significantly higher than that of all other methods (*p-values* < 0.01), which was consistent with the results of the previous study (Tan et al., 2022). Therefore, the PSO fusion method had better performance using 5 blocks of data for training and 1 block for testing. However, in the case of less training data as shown in Figure 7, the performance of the PSO fusion method was significantly lower than that of the prior fusion method. In order to reduce the calibration time, the prior fusion method was adopted in the online experiment of this study. In addition, regardless of the time cost, the ensemble task-related component analysis (e-TRCA) algorithm could be considered for EEG classification (Nakanishi et al., 2018), and PSO could be chosen as the fusion method.

**FIGURE 11:**
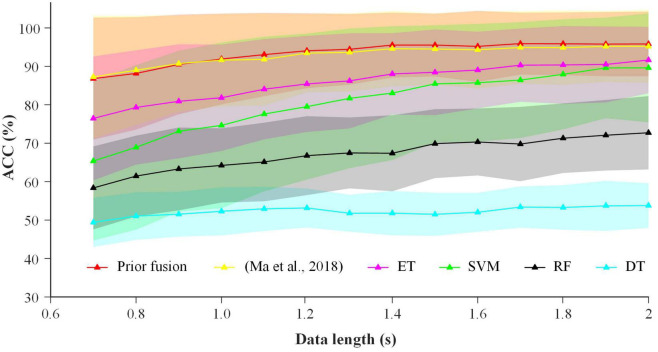
The average fusion ACCs in the stimulus length of 0.7–2.0 s with a step of 0.1 s for more different fusion methods.

In order to further illustrate the robustness of the proposed prior fusion, it was compared with the previous fusion method (Ma et al., 2018) and the common fusion algorithms including support vector machines (SVM), decision trees (DT), extreme random tree (ET) and random forests (RF). The average fusion ACCs in the stimulus length of 0.7-2.0 s with a step of 0.1 s for different fusion methods are shown in Figure 11. The results showed that the proposed prior fusion method had the best performance. Two possible reasons were explained as follows. First, the linear normalization function was used in the proposed method to convert the EEG and eye-gaze coefficients into the probability values of the same scale, which could obtain more robust classification performance. Second, the other fusion methods including SVM, DT, ET, and RF required more training data to achieve better performance, thus they had poor performance with less training trials in this study.

### 4.3. Comparison of simultaneous and sequential control modes

In this study, the simultaneous and sequential control modes were proposed. The characteristics of the two modes were compared as follows. For the simultaneous mode, the adjustable window method was used to find a suitable stimulus duration for each subject. And the data of EEG and eye-gaze were fused to classify the targets, which improved classification performance. However, system calibration was needed before the formal experiment. In addition, continuous stimulation of multiple trials could speed up the experiment, but subjects must follow the system’s fixed rhythm. And the start-stop method of eye closure and triple blink could improve the autonomy for subjects. For the sequential mode, it did not require calibration, which simplified the operation process. And partial stimulation improved classification performance and reduced subjects’ fatigue. Moreover, subjects could switch the fixation area to freely control the start of the system, which was more user-friendly. However, in order to ensure that most subjects successfully completed the control of the robotic arm, its stimulus duration was relatively long and fixed. In addition, there might be false triggers in this mode, and the execution of wrong command could be rejected by eye closure. Therefore, both the modes improved the autonomy of the subjects’ operations.

In addition, the completion time and steps of online simultaneous mode were still more than those of online sequential mode in Table 1. There were two reasons as follows. First, in the simultaneous mode, subjects were required to follow system’s fixed control rhythm. Subjects might be unable to keep up with the rhythm, resulting in incorrect commands and more completion steps. Meanwhile, in the sequential mode, subjects could think clearly before sending control commands, which reduced the number of incorrect commands. Second, it took less time to think about the same commands that were executed consecutively, and more time to think about different instructions and re-planning paths. Considering thinking time and reaction speed of different subjects, the gaze switching interval of simultaneous mode was set to 1.2 s, which might take more time. Meanwhile, the gaze switching interval of sequential mode was flexibly controlled by subjects, which could dynamically save completion time. For the above reasons, the completion time and the steps in online simultaneous mode were still more than those in online sequential mode.

### 4.4. Online mode preference and offline EEG-eye performance

**FIGURE 12:**
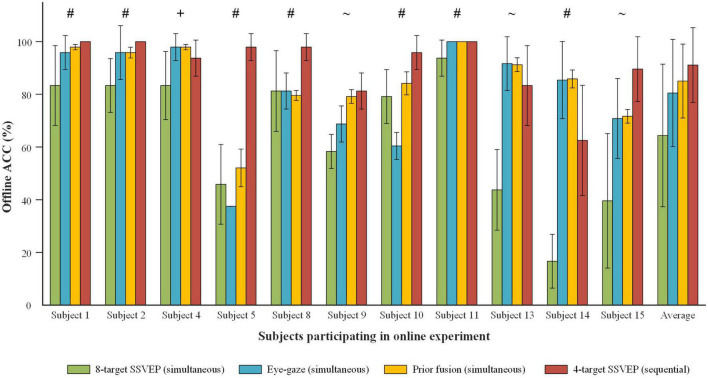
ACC comparison of 8-target SSVEP, eye-gaze and prior fusion in offline simultaneous mode and that of 4-target SSVEP in offline sequential mode at online stimulus duration for each subject who participated in online experiment. Subjects’ preference modes are expressed at the top of the bars, where ‘+’ indicates the simultaneous mode, ‘#’ indicates the sequential mode, and ‘∼’ indicates no preference.

The correlation between online mode preference and offline EEG-eye classification performance of each subject was analyzed. Figure 12 presents ACCs of 8-target SSVEP, eye-gaze and prior fusion in offline simultaneous mode and that of 4-target SSVEP in offline sequential mode at online stimulus duration for each subject who participated in online experiment. For most of the subjects, the online performance of the two modes was consistent with the offline classification performance. For Subject 4, he performed better in online simultaneous mode, and had better performance using eye-gaze than using EEG. Thus, the simultaneous mode was suggested for the subjects who were used to the control rhythm of traditional synchronous BCI and had better eye-gaze classification performance. For Subject 1, 2, 5, 8, 10, and 11, they performed better in online sequential mode, and had higher ACCs of 4-target EEG than those of prior fusion. Thus, the sequential mode was recommended for the subjects who had better EEG performance and preferred free control rhythm. In addition, Subject 9 and 13 had little difference between the EEG-eye fusion classification performance of offline simultaneous mode and the EEG classification performance of offline sequential mode, and had comparable online performance of the two modes. Subject 14 and 15 had relatively poor offline performance, which was consistent with their lower online performance. Their EEG and eye-gaze classification performance was unstable in offline and online experiments. Generally speaking, subjects’ preferred modes for online experiment were related to the offline classification performance of EEG and eye-gaze.

### 4.5. Questionnaire results

The questionnaire results of the online experiment showed that the simultaneous and sequential modes performed well in the aspects of system controllability, less fatigue of subjects, accuracy of command recognition and user-friendliness. Generally, the scores of sequential mode were higher than those of simultaneous mode, which was consistent with the online mode preference in Table 1. For the third question about concentration in the questionnaire, both the modes scored relatively low. The possible reasons for this phenomenon were explained. For the simultaneous mode, the system had a fixed gaze switching interval and subjects needed to stay focused all times. For the sequential mode, since subjects could not gaze at the stimulus areas for a long time during the thought of next command, they needed to keep their attentions to the central area. Therefore, both modes required a certain amount of concentration, which resulted in the lower scores for the third question.

### 4.6. Future work

This study provided two feasible control modes for a wearable EEG-eye-combination BCI system in VR environment. However, there is still some room for improvement in practical application. First, to explore the effect of virtual systems in rehabilitation training, it is necessary to verify the system performance for disabled persons. Second, the proposed control methods for virtual objects in VR environment can be also applied to control the real objects in AR environment. Third, a richer SSVEP stimulus method based on the virtual 3D scene can replace the traditional 2D stimulus blocks to increase the virtual experiences of the subjects. Fourth, more complex tasks can be completed by increasing the number of commands in this system.

## 5. Conclusion

In this study, an online virtual robotic arm control system was developed based on the combination of SSVEP and eye-tracking using VR head-mounted displays. The system had two control modes. For simultaneous mode, system calibration was needed before operation, and the stimulus duration of SSVEP was determined by the adjustable window method for each subject. The output command was executed according to the fusion result of EEG and eye-gaze data. In addition, triple blink and eye closure were used to control the start and stop of the system. For sequential mode, calibration was not required. Subjects could freely switch the fixation areas through eye-gaze to start the system, and then select target commands through EEG. Besides, eye closure was utilized to reject false triggering commands. The effectiveness of the proposed system was demonstrated through offline and online experiments. Moreover, the results of online experiment and questionnaire showed that both modes had better performance, but most subjects preferred the sequential mode. Comprehensively considering the convenience, efficiency and friendliness, the proposed system provided a valuable reference for the practical h-BCI system.

## Data availability statement

The raw data supporting the conclusions of this article will be made available by the authors, without undue reservation.

## Ethics statement

The studies involving human participants were reviewed and approved by Institutional Review Board of Tsinghua University. The patients/participants provided their written informed consent to participate in this study.

## Author contributions

RG completed the design and implementation of the system and data collection and analysis. RG and YL wrote the first draft. YL proposed the idea and directed the experiment. XL assisted in the debugging of the system. YL and XG provided facilities and equipment. SZ helped the complete the revised manuscript. All authors contributed to the article and approved the submitted version.
